# Feasibility Study on the Steel-Plastic Geogrid Instead of Wire Mesh for Bolt Mesh Supporting

**DOI:** 10.3390/ma15062281

**Published:** 2022-03-19

**Authors:** Qingbiao Wang, Dong Wang, Yue Li, Wenxia Liu, Chenglin Tian, Zhenyue Shi, Keyong Wang, Hongxu Song, Zhongjing Hu, Xu Zhang, Xunmei Liang, Fei Tang, Xingquan Tang, Zhengyin Liu, Mingjing Zhang

**Affiliations:** 1 College of Resources, Shandong University of Science and Technology, Tai’an 271019, China; skd990748@sdust.edu.cn (Q.W.); 150110005@sdust.edu.cn (C.T.); skd996607@sdust.edu.cn (K.W.); 2 College of Safety and Environmental Engineering (College of Safety and Emergency Management), Shandong University of Science and Technology, Qingdao 266590, China; huyang@sdust.edu.cn; 3 State Key Laboratory of Mining Disaster Prevention and Control Co-Founded by Shandong Province and the Ministry of Science and Technology, Shandong University of Science and Technology, Qingdao 266590, China; 4 National Engineering Laboratory for Coalmine Backfilling Mining, Shandong University of Science and Technology, Tai’an 271019, China; 5 College of Civil Engineering and Architecture, Shandong University of Science and Technology, Qingdao 266590, China; 201982040033@sdust.edu.cn (D.W.); 201983040032@sdust.edu.cn (Y.L.); 201982040021@sdust.edu.cn (X.Z.); 6 Mountain Tai Composite Industry Research Institute, Tai’an 271019, China; liuwenxia@163.com; 7 College of Energy and Mining Engineering, Shandong University of Science and Technology, Qingdao 266590, China; 201983010016@sdust.edu.cn; 8 Shandong Road New Materials Co., Ltd., Tai’an 271019, China; ludelxm@163.com; 9 Shandong Luda Test Instrument Co., Ltd., Tai’an 271019, China; tangfei88@vip.163.com (F.T.); xingquan516@163.com (X.T.); 10 Shandong Provincial Communications Planning and Design Institute Group Co., Ltd., Jinan 250031, China; liuzhengyin009@126.com (Z.L.); sdjtyyt@126.com (M.Z.)

**Keywords:** steel-plastic geogrid, wire mesh, bolt mesh supporting, mesh wire tensile testing, node peel test, mesh load-bearing capacity test, feasibility studies

## Abstract

Wire mesh is a common material for bolt mesh supporting structures, but its application in engineering has revealed many defects. At the same time, with the development of new materials for civil engineering, the new material mesh performance and cost show outstanding advantages over wire mesh. In this paper, the feasibility of replacing wire mesh with steel-plastic geogrid as an alternative material is carefully studied through indoor tests and field applications. The following conclusions were drawn from a comparative analysis with wire mesh, mainly in terms of mechanical properties, engineering characteristics, and construction techniques: (1) in terms of mesh wire strength, wire mesh is slightly better than steel-plastic geogrid, but in the case of similar tensile strength, the amount of steel used per unit length of steel geogrid bars is only 36.75% of that of steel-plastic geogrid, while the tensile strength of the high-strength steel wire attached to the steel-plastic geogrid belt is about 3.3 times that of steel bars; (2) in terms of junction peel strength, both values are similar, with the injection-moulded junction being 1154.56–1224.38 N and the welded junction of 4 mm mesh being 988.35 N; (3) in terms of the strength of the mesh, steel-plastic geogrid is better than wire mesh, and with the same mesh wire strength, the bearing capacity of steel-plastic geogrid is increased by about 63.17% and the contribution of the mesh wire bearing capacity is increased by 83.66%, with the damage mainly being in the form of wire breakage in the ribbon causing ribbon failure, leading to further damage to the mesh; (4) in terms of the engineering application of steel-plastic geogrid compared to wire mesh, the utilization rate of mesh increases by about 24.99%, the construction efficiency increases by about 14.10%, and the economic benefit increases by about 45.31%. In practical application, the steel-plastic geogrid has good adhesion with surrounding rock and strong corrosion resistance. According to the above research analysis, the steel-plastic geogrid is feasible to replace the wire mesh for bolt mesh supporting.

## 1. Introduction

Bolt mesh supporting systems have gradually become the most common method used in underground coal mines around the world. In China, 80% of newly excavated tunnels are supported by anchor mesh [1]. As an important part of the bolt mesh supporting structure, the mesh plays an indispensable and important role. Metal mesh has been widely used as the main mesh material for the support of roadways, tunnels, and slope stabilization projects [2,3]. However, as the research progressed, many problems were revealed in various aspects of the metal mesh, mainly reflected in easy corrosion, low junction strength, low construction efficiency, poor integrity, low strength utilization, etc. [4,5].

Scholzen, A. et al. [6] and Curbach, M. et al. [7] have responded to the problems that have risen with metal mesh. The performance of carbon fibres (CF) reinforcement with high corrosion resistance has been investigated separately for application in concrete structures. Mechtcherine, V. et al. [8] investigated a mineral-impregnated carbon fibre composites (MCF) reinforcement to improve the high-temperature resistance based on the inherited corrosion resistance of (CF) reinforcement and proposed a continuous robotic-based structuring process for the production of the reinforcement mesh. Yang, X. et al. [9] proposed a new “shell + bolt + shotcrete” combined support structure and verified its feasibility by numerical simulation and model test. Shan, Z.J. et al. [10] proposed the use of thin spray-on liner (TSL) material as an alternative to reinforcing mesh for mine support. Liang, H. et al. [11] investigated the performance of a reinforcing TSL material on this basis. Xu, C. et al. [12] studied the discrete element model between metal mesh and rock, simulated more than 900 times, and studied the influence of various factors on mesh deformation. Yuan, G.Y. et al. [13] obtained the supporting strength, stress–strain distribution, and force transmission measurement of metal mesh under vertical load by static load test and numerical simulation and proposed corresponding reinforcement measures for different metal mesh. Dale, K. [14] proposed a new “kNotted” type of metal mesh that can increase the load-bearing capacity of the mesh by approximately 50%. 

Ding, Y.N. et al. [15] explored the feasibility of blending steel and glass fibre mesh as an alternative to conventional reinforcing mesh. Mujah, D. et al. [16] investigated the potential use of three-dimensional geomaterials (8FG MAT) derived from glass fibres recovered from industrial glass fibre waste. Qazia, S. et al. [17] studied a new anchor mesh material consisting of carbon fibre-reinforced plastics. Miskolczi, N. [18] studied the reliability of fibre-reinforced polymer (FRP) composites as anchor mesh materials.

The above research provides broad ideas for our research in the field of reinforcing mesh and anchor mesh structures for their own load-bearing capacity enhancement, as well as new materials and structures.

**Figure 1:**
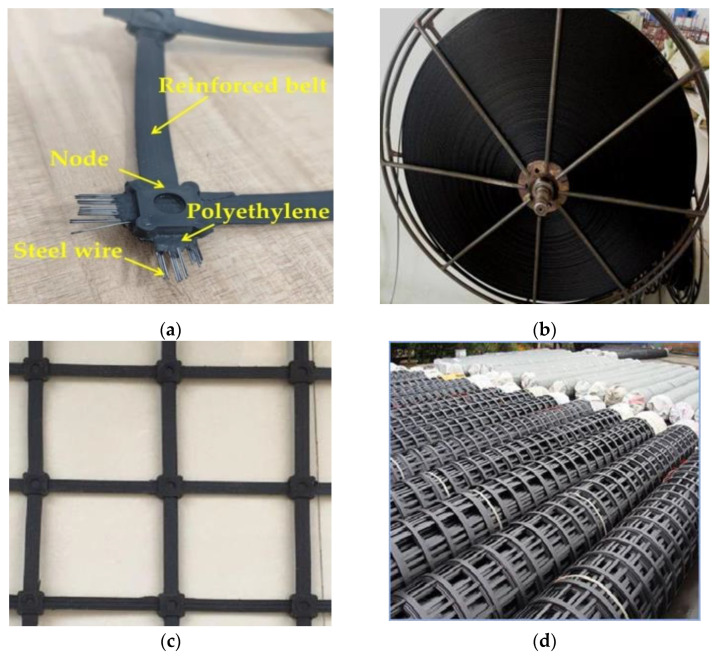
Steel-plastic geogrid structural structure and finished product display. (**a**) Steel-plastic geogrid ribbon construction; (**b**) finished steel-plastic geogrid reinforced belt; (**c**) steel-plastic geogrid mesh; (**d**) steel-plastic geogrid rolls.

The steel-plastic geogrid is a new type of geocomposite material, made by extruding a high-strength steel wire as the inner skeleton wrapped with a high-strength polyethylene protective layer and adding relevant additives, and then using injection bonding technology to weld the ribbon to make a steel-plastic geogrid mesh, as shown in Figure 1. Steel-plastic geogrid has the advantages of good corrosion resistance, high load-bearing capacity, high junction strength, good flexibility, and strong integrity. Numerous scholars have studied its properties and applications: Nakamura, T. et al. [19] investigated the deformation characteristics of steel-plastic geogrid by means of straight shear tests. Wang, Q.B. et al. [20] investigated the tensile strength and deflection of steel-plastic geogrid through tests. M. G. Hussein et al. [21] proposed a procedure for the 3D finite element analysis of unconfined and soil-confined geogrid developed using ABAQUS software and verified its effectiveness in capturing a three-dimensional response. The reinforcement capacity of steel-plastic geogrid in roadbeds has been studied by scholars [22,23,24] such as Matveev, S.A. et al. [25], and Tang, T. [26]. Wang, C. et al. [27] and Xiao, C.Z. [28] investigated the application of steel-plastic geogrid in the reinforcement of retaining walls, and Du, K.Q. et al. [29] studied the reinforced friction mosaic occlusion performance of monolithic steel-plastic geogrid to provide theoretical guidance for slope management. He, Y.F. et al. [30] investigated the reinforcing effect of steel-plastic geogrid in copper mine tailings fills.

**Figure 2:**
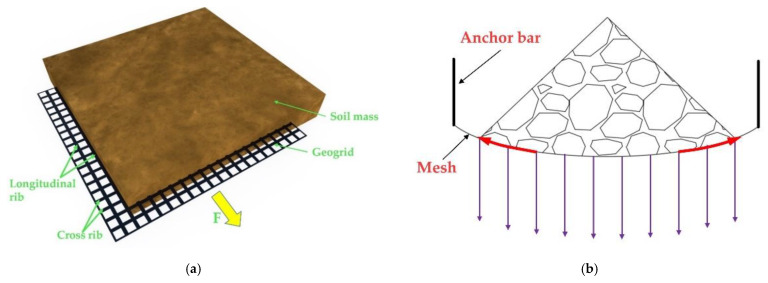
Mechanism of geogrid in different fields: (**a**) reinforcement field; (**b**) bolt mesh supporting field.

Previous research on steel-plastic geogrid has focused on the reinforcement of road foundations, retaining walls, slopes, fills, and other areas. The study of the reinforcement mechanism has been relatively mature, but the application and study of steel-plastic geogrid in the field of bolt mesh supporting has not yet been carried out systematically. The reinforcement mechanism of the geogrid is not the same as that of the bolt mesh supporting. The reinforcement of the soil is mainly based on the surface friction between the soil and the geogrid and the bite force between the cross ribs of the geogrid and the soil (Figure 2a); the support of the surrounding rock is mainly based on the tensile strength to withstand the load from the geotechnical body (Figure 2b). The high-strength steel wire added in the steel-plastic geogrid has superior tensile properties, which makes it different from the ordinary geogrid [31]. It has better tensile and bearing properties and great potential in the field of bolt mesh supporting. Therefore, it is necessary to systematically study the application of steel-plastic geogrid in the field of anchor networks.

In order to achieve the objective of replacing wire mesh with steel-plastic geogrid, it was first necessary to compare and analyze the possibility of replacing wire mesh. Therefore, this paper compares the load-bearing performance of wire mesh and steel-plastic geogrid through systematic tests and applies steel-plastic geogrid to the actual bolt mesh supporting construction. The application of steel-plastic geogrid instead of wire mesh in bolt mesh supporting is systematically studied in terms of the construction process, construction time efficiency, and economic benefits, and the feasibility of steel-plastic geogrid instead of wire mesh for bolt mesh supporting are explored to provide a theoretical basis for the future application of steel-plastic geogrid in bolt mesh supporting projects.

## 2. Steel-Plastic Geogrid Versus Steel Wire Mesh

### 2.1. Comparison of the Tensile Properties of the Mesh Wire

#### 2.1.1. Experimental Programme Design

**Figure 3:**
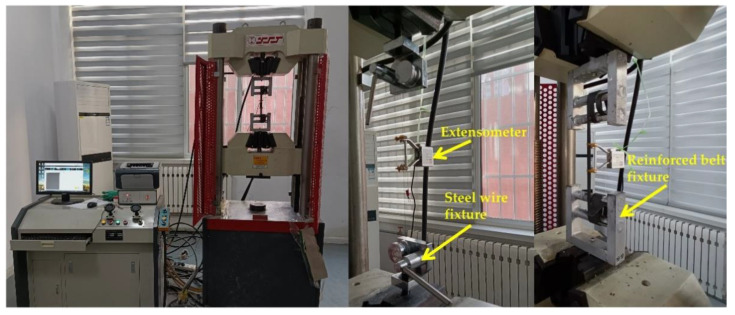
Test equipment and exclusive fixtures.

The main reliance on the performance of the net was on the tensile properties of the mesh wire, so these were tested first. The wire mesh types commonly used in underground projects such as mines and tunnels were selected and their performance tests with steel-plastic geogrid mesh wire were carried out to analyse the raw material properties and characteristics. The stiffness of the reinforcing mesh increased with the diameter of the wire, and too much stiffness made it difficult to fit the mesh to the surrounding rock. Therefore, the most commonly used 4 mm and 6 mm sizes of wire mesh were selected for support, and an additional 8 mm size was added for comparison. The wire mesh wire was cold drawn steel, so for the steel and steel-plastic geogrid bar tape tensile test, the test was divided into three groups, with each group setting up six identical samples. The length of the material selected was 25–30 cm, the test results were taken as the average of each group test result, and the detailed test group design is shown in Table 1. The test machine was a microcomputer-controlled electro-hydraulic servo universal testing machine, and the high-strength steel wire and steel-plastic grille bar tape were tested using the corresponding winding-type fixture model. The load sensor was the tension sensor of the universal testing machine, which can automatically collect real-time load. The stress and strain test device was the YYU-5/50 extensometer, produced by the Iron and Steel Research Institute, as shown in Figure 3.

#### 2.1.2. Experimental Programme Design

**Figure 4:**
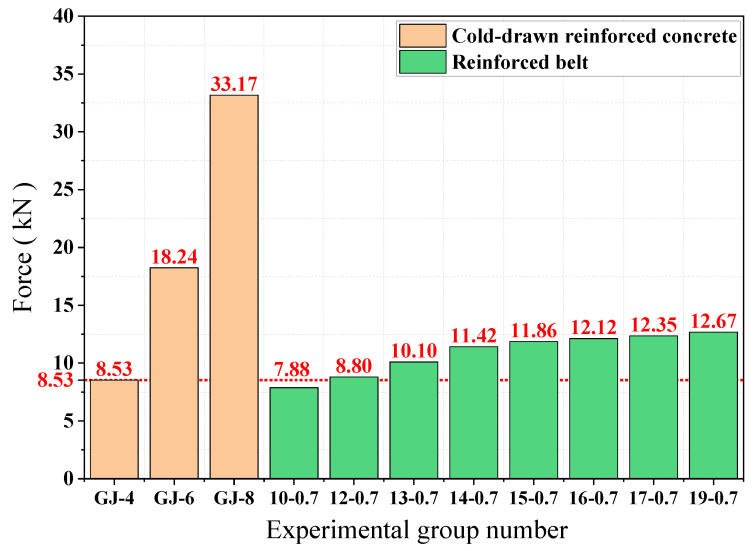
Comparison of peak mesh wire tension.

As can be seen from Figure 4, the peak tensile forces of GJ-4, GJ-6, and GJ-8 cold-drawn bars are 8.53 KN, 18.24 KN, and 33.17 KN, respectively, and the peak stresses increase as the diameter of the bars increases. The peak tensile forces of the 10-0.7, 12-0.7, 13-0.7, 14-0.7, 15-0.7, 16-0.7, 17-0.7, and 19-0.7 steel-plastic geogrid-reinforced belt were 7.88 KN, 8.80 KN, 10.10 KN, 11.42 KN, 11.86 KN, 12.12 KN, 12.35 KN, and 12.67 KN. With the increase in the number of high-strength steel wires attached, the peak stress of the band tends to increase. The peak tension values for the steel reinforcement bars of type GJ-4 and the steel-plastic geogrid bars of type 12-0.7 are similar, at 8.53 KN and 8.80 KN, respectively. The overall peak tensile strength of the wire mesh wire was greater than that of the steel-plastic geogrid wire. In terms of the amount of steel used per unit length, as shown in Table 1, the experimental group GJ-4 was 12.57 mm^3^ and the experimental group 12-0.7 was 4.62 mm^3^. The peak tensile strength of the two test groups was similar, but the amount of steel used per unit length of the steel-plastic geogrid reinforced belt was 36.75% of that of the steel bar.

**Figure 5:**
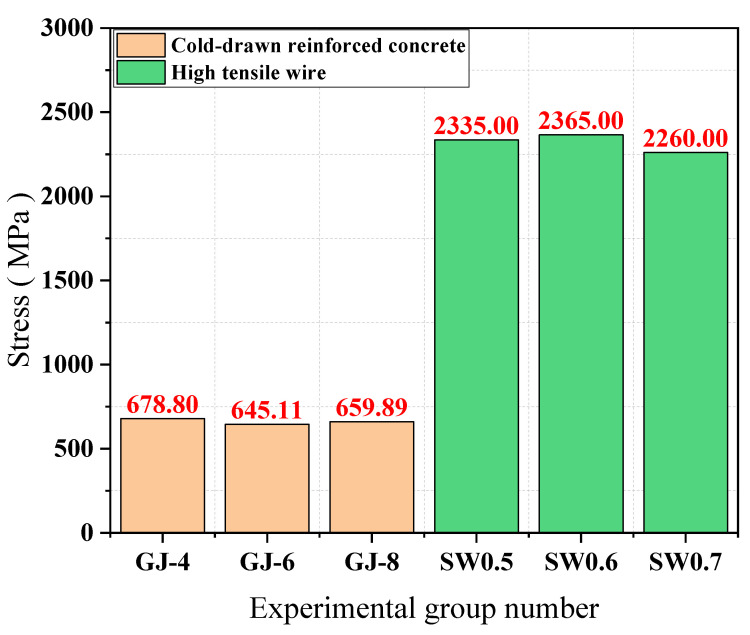
Comparison of peak wire stresses.

The stress is calculated using Equation σ=N/S, where σ is the tensile stress, *N* is the tensile force, and *S* is the initial area of the specimen. The results of the calculation are shown in Figure 5. As seen in Figure 5, the peak stress values of the wire mesh wire remain consistent, reaching 645.11~678.80 MPa; the peak stress of the high-strength wire also remains consistent, reaching 2260.00~2360.00 MPa; the peak stress of the high-strength wire is about 3.3 times higher than the peak stress of the wire mesh wire, indicating that the tensile strength of the high-strength wire is higher.

### 2.2. Comparison of Nodal Strengths

#### 2.2.1. Experimental Programme Design

**Figure 6:**
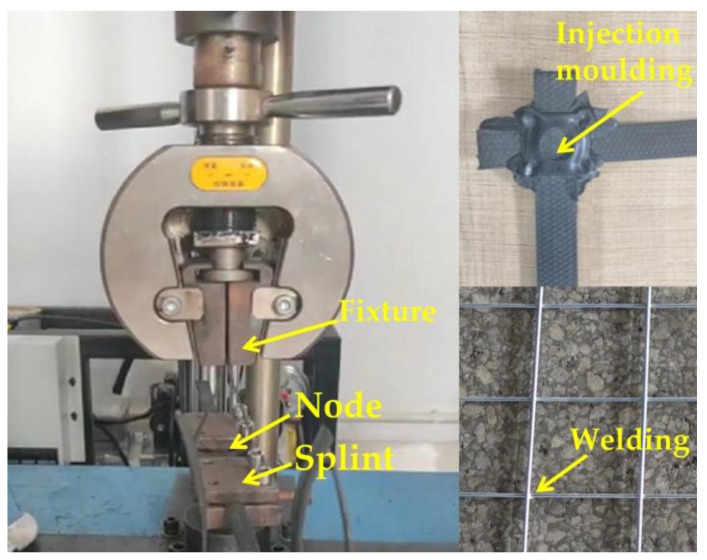
Test demonstrations.

The nodes are the key to joining the mesh wire into a mesh, and as there have been many instances of mesh destabilisation due to node damage in relevant tests, tests on the peeling properties of the nodes should be carried out. Welded joints commonly used for steel mesh and injection-moulded joints commonly used for steel-plastic geogrid were selected for testing. Some of the same types of wire were selected for the connection and the experimental group design is shown in Table 2. A peel test was carried out, as shown in Figure 6.

#### 2.2.2. Analysis of Test Results

**Figure 7:**
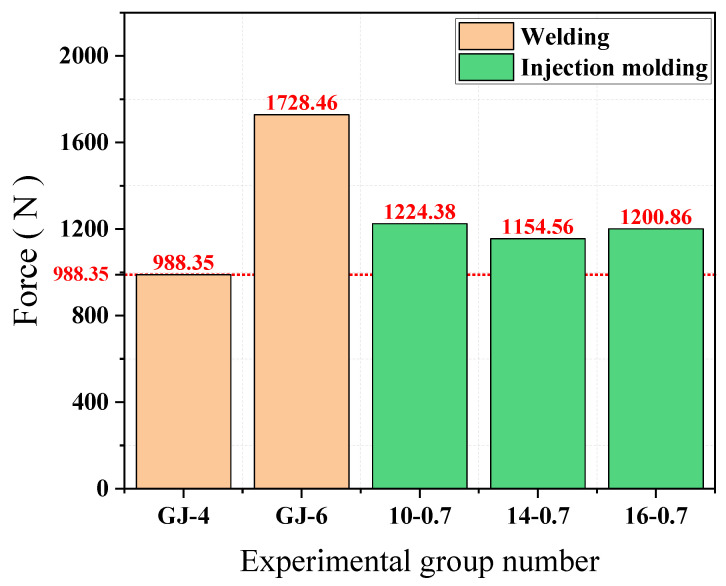
Comparison of node strength by test group.

As can be seen from Figure 7, the peeling force of the welded joints of GJ-4 bars is 988.35 N and the peeling force of the welded joints of GJ-6 bars is 1728.46 N, with the increasing diameter of the bars the peeling; the peeling force of the injection-moulded joints remains the same, reaching 1154.56–1224.38 N. The peel force of injection-moulded joints is less than that of welded joints type GJ-6 and is similar to that of welded joints type GJ-4. In practice, welded joints are prone to corrosion, leading to a reduction in the strength of the joints and the peel force of the joints.

### 2.3. Comparison of Mesh Load-Bearing Capacity

#### 2.3.1. Experimental Programme Design

**Figure 8:**
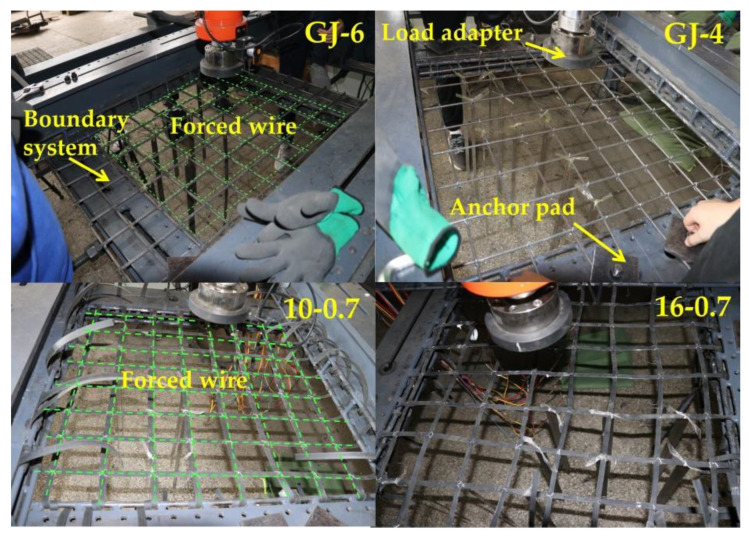
Partial specimen mounting.

**Figure 9:**
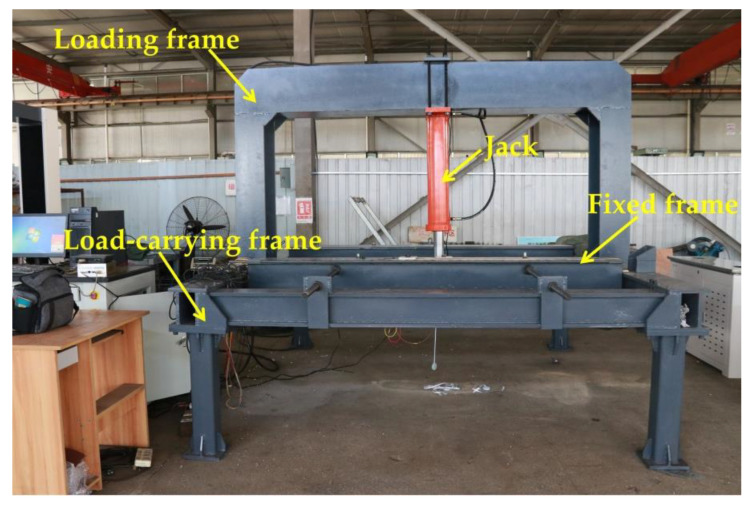
Net performance testing testbed.

The same type of mesh wire was connected to form the mesh to ensure that the material parameters of the wire mesh, as well as the steel-plastic geogrid wire, were exactly the same as those used in the wire tests for each test group, as shown in Table 3. Some of the specimens are shown in Figure 8. The test machine used an independently developed test platform for testing the performance of the nets, Figure 9.

#### 2.3.2. Analysis of Test Results

**Figure 10:**
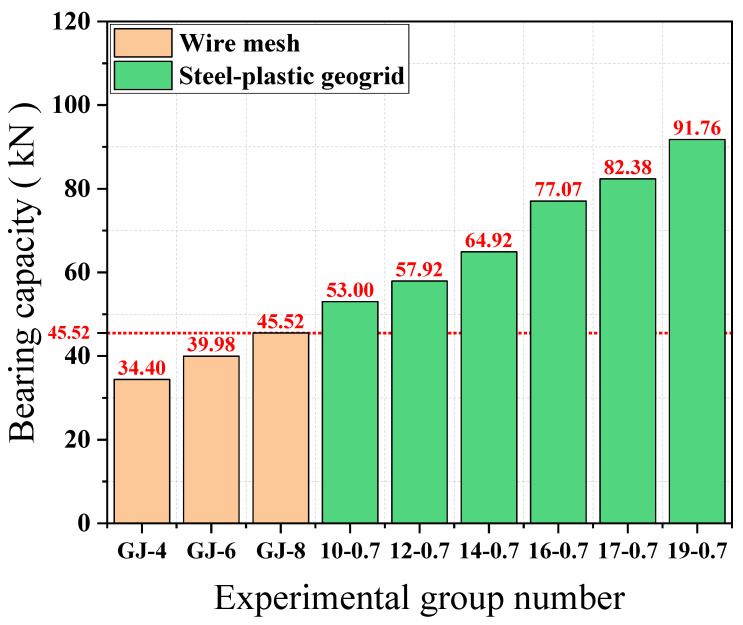
Comparison of peak mesh load-bearing capacity.

From Figure 10, it can be seen that the load-bearing capacity of the wire mesh of the GJ-4, GJ-6, and GJ-8 models are 34.40 KN, 39.98 KN, and 45.52 KN, respectively, with the increase of mesh wire diameter and the load-bearing capacity of wire mesh showing an increasing trend. The load-carrying capacity of the steel plastic geogrid with models 10-0.7, 02-0.7, 14-0.7, 16-0.7, 17-0.7, and 19-0.7 is 53.00 KN, 57.92 KN, 64.97 KN, 77.0 7 KN, 82.38 KN, and 91.76 KN, respectively, with the increase of the number of steel wires attached to the mesh and the load-carrying capacity of the steel-plastic geogrid showing an increasing trend. The peak tensile strength of GJ-4 reinforcement and 12-0.7 reinforcement is similar in the mesh wire experiment, but the corresponding mesh bearing capacity in the net bearing capacity experiment is larger, at 34.40 KN and 57.92 KN, respectively, and the bearing capacity of steel-plastic geogrid is improved by about 83.66% compared with wire mesh. This means that the mesh is a whole structure and the load-bearing capacity of the mesh wire is only one factor affecting the load-bearing capacity of the mesh. The overall load-bearing capacity of the steel mesh is less than that of the steel-plastic geogrid, indicating that the steel-plastic geogrid is superior to the steel mesh in terms of the load-bearing capacity of the mesh.

**Figure 11:**
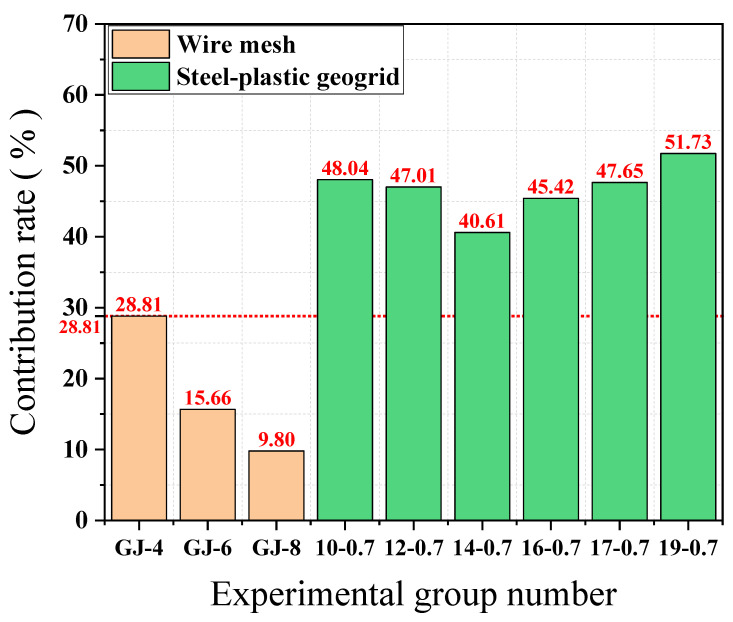
Contribution of mesh wire carrying capacity.

Figure 11 shows the ratio of the average tension of each mesh wire in the mesh test to the tension in the individual mesh wire test. The contribution of mesh wire load-bearing capacity of GJ-4, GJ-6, and GJ-8 models are 28.81%, 15.66%, and 9.80%, respectively, and the contribution of the wire load-bearing capacity of the wire mesh is decreasing with the increase of the diameter. The contribution of the 10-0.7, 12-0.7, 14-0.7, 16-0.7, 17-0.7, and 19-0.7 mesh wires is 48.04%, 47.01%, 40.61%, 45.42%, 47.65%, and 51.73%, respectively, with the increase of the number of high tensile steel wires attached and the contribution of the mesh wire to the load-bearing capacity decreasing and then increasing. The contribution of the 14-0.7-type wire is the smallest, with a minimum value of 40.61%. The peak tensile strengths of the GJ-4 and 12-0.7 bars are similar in the mesh wire experiments, but the difference in the contribution of the mesh wire is large, at 28.81% and 47.01%, respectively, and the contribution of the steel-plastic geogrid compared to the steel mesh is increased by about 63.17%. The contribution of geogrid mesh wire is greater than that of wire mesh, indicating that steel-plastic geogrid is superior to wire mesh in terms of mesh wire load capacity utilisation. Although the peak tensile strength of the wire mesh wire is better than that of the steel-plastic geogrid wire, the mesh is a structure composed of wire, and since the wire mesh fails to give full play to the overall performance of the structure, this results in a waste of wire strength and a lower overall load-bearing capacity of the mesh.

**Figure 12:**
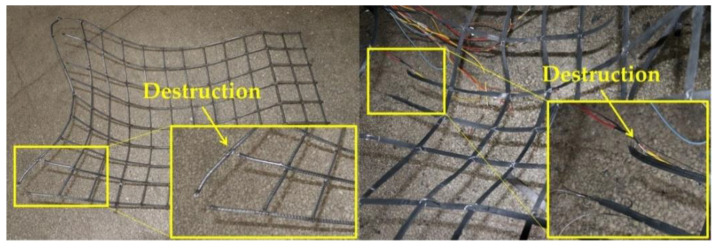
Diagram of mesh damage.

As can be seen from the enlarged view of the damage at the bottom right of Figure 12, the damage to the wire mesh occurred at the knots and was a failure of the mesh due to damage to the knots. The damage to the steel-plastic geogrid was the failure of the mesh caused by the pulling and drawing of the high-strength wire in the tendon band, indicating that the strength of the wire in the wire mesh was not fully utilised, corresponding to the small utilisation rate and the contribution of the wire mesh wire to the bearing capacity of the wire mesh in the previous section.

In summary, steel-plastic geogrid is superior to wire mesh in terms of load-bearing capacity and the contribution of the mesh wire to the load-bearing capacity. Therefore, in terms of mechanical properties, steel-plastic geogrid has the possibility of replacing wire mesh for containment.

### 2.4. Summary of Indoor Tests

In order to visualise the mechanical properties of steel-plastic geogrids and metal mesh, the important data from the mesh wire tensile test, knot peel test, and mesh bearing capacity test are summarised in Table 4.

It can be seen from Table 1 that when the strength of the steel-plastic geogrid is similar to that of the metal mesh wire, the steel-plastic geogrid has less steel consumption per unit length, high strength of node peeling force, good network-bearing capacity, and a high contribution rate of network wire-bearing capacity. At the same time, the peak bearing capacity of the steel-plastic geogrid is higher than that of the metal mesh in the experiment of network-bearing capacity.

### 2.5. Engineering Tests

#### 2.5.1. Construction Process

The tests were carried out in the Xiaogou mine in Yiyuan County, Shandong Province, where wire mesh and steel-plastic geogrid were selected as the main mesh materials for bolt mesh supporting. The construction processes of the two mesh materials were compared, the similarities and differences in the process were summarised, and the corresponding test data were collected.

**Figure 13:**
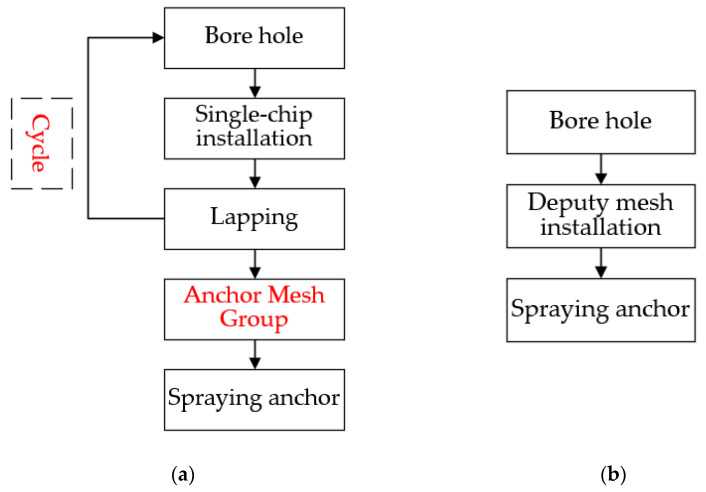
Site hanging construction drawings: (**a**) wire mesh; (**b**) steel-plastic geogrid.

**Figure 14:**
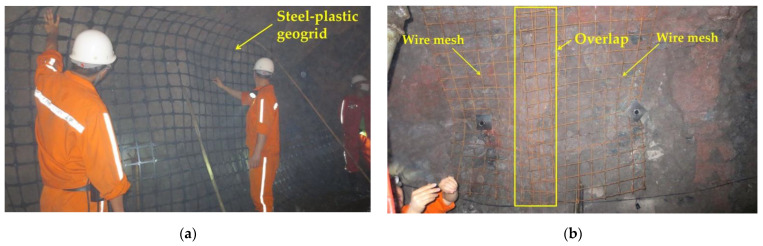
Comparison of on-site construction processes: (**a**) steel-plastic geogrid; (**b**) wire mesh.

**Figure 15:**
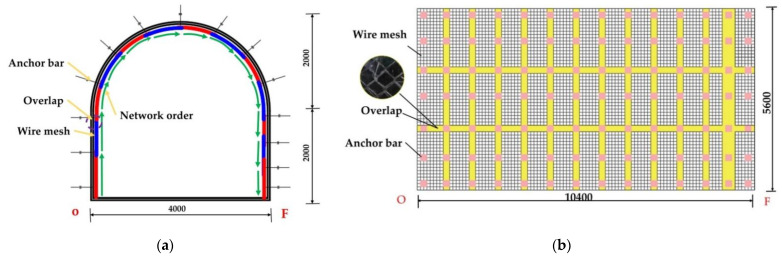
Construction diagram of wire mesh laying: (**a**) schematic diagram of the profile; (**b**) schematic diagram of section unfolding.

The anchor network support is a common support method in underground structures such as galleries and tunnels. The composite body formed by anchor rods, anchor networks, and the surrounding rock makes up for the lack of strength of the surrounding rock itself. Whether to choose shotcrete for further reinforcement of the surrounding rock needs to be decided according to the actual situation on site. Figure 13 shows the construction process of the bolt mesh supporting during one construction of the roadway section without the lap construction of the mesh in the direction of the roadway depth. The anchor mesh construction process is similar for both types of mesh, the wire mesh needs to be looped and lapped to form an anchor mesh group, as in Figure 14a, and the steel-plastic geogrid needs to be installed only once, as in Figure 15a. There are differences in the hanging process.

#### 2.5.2. Comparison of Hanging Processes

**Figure 16:**
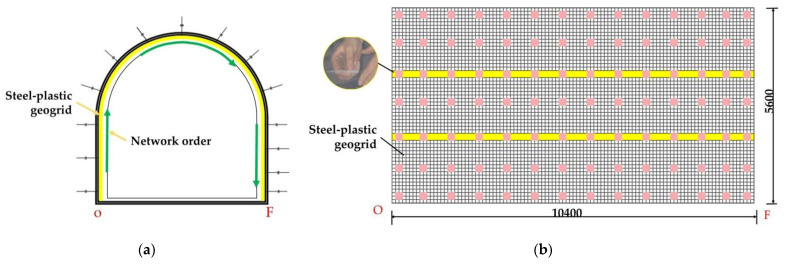
Diagram of steel-plastic geogrid laying construction: (**a**) schematic diagram of the profile; (**b**) schematic diagram of section unfolding.

From Figure 14, it can be seen that the steel-plastic geogrid sheet has a large width and good flexibility, while the steel mesh sheet has a small width and poor flexibility. The steel-plastic geogrid is hung in full section: (1) the end-up hanging method, as in Figure 15a, i.e., the starting point of the hanging network is located at the bottom corner of the tunnel and is laid along the surrounding rock towards the other bottom corner; (2) the middle start method, where the starting point of the netting is located in the centre line at the top of the lane and is laid along towards the two bottom corners. The wire mesh is laid along the surrounding rock at a strictly required starting point at one of the bottom corners, as in Figure 16a. The full section hanging process reduces the number of laps compared to reinforced steel mesh, increasing construction efficiency and saving costs.

The lapping process uses a 10 * 450 nylon tie (single tension: approx. 114 kg) to lap the steel-plastic geogrid, as shown in Figure 15a. The wire mesh is lapped with 14# wire (single tensile strength approx. 110 kg) as shown in Figure 16b. Compared to wire, nylon ties are easier to handle, increase efficiency, and save labour costs when the tension is similar.

Figure 15a shows a section of a semi-circular arched reinforced mesh support on-site, with a section height of 4 m, width of 4 m, wall height of 2 m, and arch height of 2 m. Figure 14b shows the expansion diagram of the bolt mesh supporting starting from point O, along the direction of mesh laying, with a support length of 10.4 m and a supported depth of 5.6 m. Figure 15 shows the schematic diagram of the steel-plastic grid support under the same dimensions. Table 5 shows the material statistics in the entire test support spread. As can be seen from Figure 16, the steel mesh support requires several laps to form the anchor mesh cluster, while the steel plastic grid requires only a few laps. As can be seen from Table 5, the average anchor use for the same support area is the same for both steel-plastic geogrid and wire mesh, both at 1.96 /m^2^. The utilisation rate of the mesh is 93.33% for steel-plastic geogrid and 74.67% for reinforced steel mesh—an increase of about 24.99% compared to reinforced steel mesh. The lap area ratio is 7.14% for steel-plastic geogrid and 30.36% for steel wire mesh, which is about four times more than steel-plastic geogrid. Compared with steel wire mesh, steel-plastic geogrid has fewer laps and good integrity under the same average usage of anchor rods, small lap area as a percentage of good integrity, easy construction, high construction efficiency, high utilization rate of mesh, large effective utilization area of the mesh, material saving, and cost-saving.

#### 2.5.3. Comparison of Hanging Efficiency

The results of the observations on-site and the survey of construction personnel, which counted the time spent on the construction of the individual hanging screens and were collated, are shown in Table 6.

As can be seen from Table 6, the time spent in drilling and setting anchors for steel-plastic geogrids and wire mesh is the same, at 8 min/each and 1.5 min/each. The time used for laying the mesh is 0.5 min/m^2^ for steel-plastic geogrid and 1 min/m^2^ for steel wire mesh, which is smaller than steel wire mesh because steel-plastic geogrid, with its larger mesh, is lighter and has fewer handling times. The average lap time used is 5 min/m^2^ for steel-plastic geogrids and 6.5 min/m^2^ for reinforced steel mesh. Steel-plastic geogrids are smaller than reinforced steel mesh because they have fewer laps, which reduces the number of machine stops, and they are easy to construct with nylon tie laps. There is an average hanging time of 16.88 min/m^2^ for steel-plastic geogrids and 19.30 min/m^2^ for wire mesh, an increase of about 14.10% in hanging efficiency. Steel-plastic geogrids take less time to hang than steel-plastic geogrids, which shows that steel-plastic geogrids are more efficient than steel mesh in hanging construction.

#### 2.5.4. Comparative Economic Benefits

The cost of the test section was calculated by investigating the cost of materials and labour at the construction site, combined with market research, and the results are shown in Table 7.

As can be seen from Table 7, the average cost of steel-plastic geogrid is 80.23 /m^2^ and wire mesh is 116.58 /m^2^. The cost per square metre of steel-plastic grating is approximately 45.31% less than that of wire mesh, which means that the cost of steel-plastic grating is lower and more economical than wire mesh.

#### 2.5.5. Comparison of Other Engineering Features

**Figure 17:**
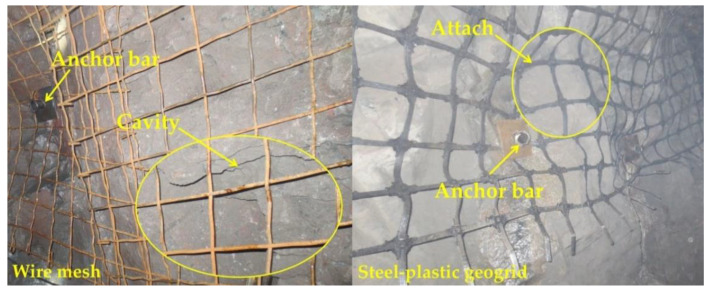
Comparison of the degree of fit of the mesh to the surrounding rock.

As can be seen from Figure 17, the steel-plastic geogrid has a better fit to the surrounding rock and a higher integrity compared to the steel wire mesh. The wire mesh is a flat structure and since the shape of the surrounding rock at the lap is usually irregular, this makes it difficult to ensure the simultaneous deformation of the anchor mesh and the surrounding rock. When the surrounding rock is deformed, the load acts directly on the wire mesh, which is then transferred to the anchor rods, resulting in the anchor rods being susceptible to failure due to excessive force, which in turn leads to instability of the surrounding rock. The deformation capacity of the steel-plastic geogrid is good. When subjected to a large surrounding rock force, a certain degree of “pressure deformation” is carried out first, releasing a certain amount of energy, after which the force transferred to the anchor rod is reduced, making the anchor rod less prone to instability and good support stability.

**Figure 18:**
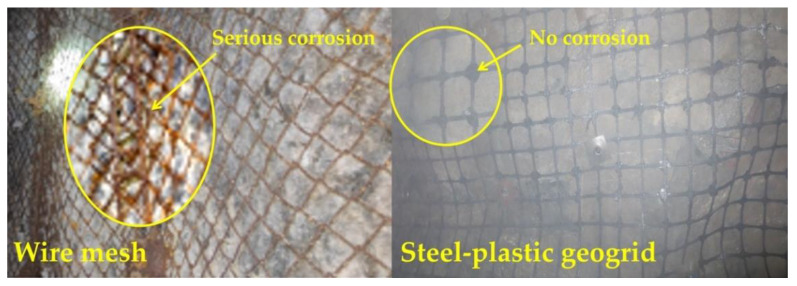
Comparison of mesh erosion and corrosion.

As can be seen in Figure 18, the wire mesh has undergone severe rusting due to prolonged exposure. The polyethylene outer layer of the steel-plastic geogrid has excellent corrosion protection and encases the rigid steel wire, effectively preventing corrosion of the wire and extending the life of the steel-plastic geogrid.

### 2.6. Summary of Engineering Tests

In order to visualise the application of steel-plastic geogrids and metal mesh in engineering, the key data from engineering tests are summarised in Table 8.

As can be seen from Table 8, the steel-plastic geogrid has a higher utilization rate, less lap area, lower construction time, and lower construction costs than metal mesh in the more important projects in the anchor net construction process. Therefore, in terms of practical engineering performance, it is feasible to use steel-plastic geogrids instead of metal mesh for anchor mesh construction.

## 3. Discussion

In this paper, in order to study the feasibility of replacing steel wire mesh with steel-plastic geogrid in support, tests on mesh wire, knots, and nets were carried out and combined with test comparisons in the field, and the feasibility of replacing steel wire mesh with steel-plastic geogrid for bolt mesh supporting was studied. However, a systematic study is needed for the promotion and application of steel-plastic geogrid.

The load-bearing properties of steel-plastic geogrids can vary with different mesh wire strengths, sizes, and surface areas, so it is essential to further investigate the specific effects of these factors on the load-bearing capacity of steel-plastic geogrids.

At this stage, the steel-plastic geogrid for anchor network support has not yet formed a relevant, systematic supporting construction technology, while the corresponding production, the installation apparatus development work, has not yet been fully developed. It is also necessary to improve and upgrade the steel-plastic geogrid products to prevent them from being replaced due to the backwardness of the products.

## 4. Results

Based on the tensile test of the mesh wire and the peeling test of the knots, the load-bearing capacity test of the wire mesh and the steel-plastic geogrid was carried out, the contribution value of the mesh wire load-bearing was calculated, and the construction efficiency, economic efficiency, and other engineering construction priorities of both were studied in conjunction with the corresponding engineering construction, and the following conclusions were obtained.

(1) Wire mesh is slightly superior to steel-plastic geogrid in terms of mesh wire strength. However, the amount of steel used for the similar mesh wire strength of the steel-plastic geogrid bars is less than that of the wire mesh, while the tensile strength of the high-strength steel wires attached to the bars is much greater than that of the steel bars.
(2) In terms of junction peel strength, the injection-moulded junction peel force is similar to that of a 4 mm diameter wire mesh-welded junction.
(3) In terms of the strength of the mesh, the load-bearing capacity of steel-plastic geogrid is better than that of wire mesh. The load-bearing capacity of steel-plastic geogrids is far better than that of wire mesh at similar mesh wire strengths, and the performance of steel-plastic geogrid mesh wire is excellent. The steel-plastic geogrid outperforms the steel wire mesh in terms of the contribution of the mesh wire load capacity, again indicating that the steel-plastic geogrid is better at reusing the mesh wire load capacity. In terms of damage, most of the wire mesh fails due to junctional damage, and most of the steel-plastic geogrids fail due to wire pull-out damage in the reinforcement band.
(4) In terms of engineering application, steel-plastic geogrid is more suitable for anchor net support construction as it has fewer laps than steel mesh, high mesh utilisation, high construction efficiency, good economic benefits, high degree of adhesion to the surrounding rock, good integrity, high strength, and high corrosion resistance.

In summary, steel-plastic geogrids are feasible in terms of mechanical properties, construction efficiency, and economic benefits in place of steel wire mesh for bolt mesh supporting.

## Figures and Tables

**Table 1:** Mesh wire test group design table.

Type of Material	Test Group Number	Number of Wires (rods)	Wire Diameter (mm)	Cross-Sectional Area (mm^2^)	Amount of Steel Used Per Unit Length (mm^3^)
High tensile steel wire	SW0.5	/	0.5	0.20	0.20
	SW0.6	/	0.6	0.28	0.28
	SW0.7 *	/	0.7	0.38	0.38
Cold-drawn steel bars	GJ-4 *	/	4	12.57	12.57
	GJ-6	/	6	28.27	28.27
	GJ-8	/	8	50.27	50.27
Steel-plastic geogrid reinforced belt	10-0.7 *	10	0.7	38.94	3.85
	12-0.7	12		30.14	4.62
	13-0.7	13		33.44	5.00
	14-0.7	14		41.13	5.39
	15-0.7	15		39.48	5.77
	16-0.7	16		36.76	6.16
	17-0.7	17		33.12	6.54
	19-0.7	19		33.12	7.31

* 10-0.7 denotes steel-plastic geogrid reinforced belt with 10 high tensile steel wires with a diameter of 0.7 mm; GJ-4 denotes cold-drawn low carbon steel bars with a diameter of 4 mm; SW-0.7 denotes high tensile wire with a diameter of 0.7 mm.

**Table 2:** Peel test group design.

Type of Material	Test Group Number	Connection Method
Cold-drawn steel bars	GJ-4	Welding
	GJ-6	
Steel-plastic geogrid reinforced belt	10-0.7	Injection-moulding
	14-0.7	
	16-0.7	

**Table 3:** Design of test sets for mesh load-bearing capacity tests.

Type of Material	Test Group Number	Mesh Size (mm)	Grid Size (mm)	Actual Number of Mesh Wires Carried
Steel-plastic geogrid	10-0.7 *	800 × 800	100 × 100	14
	12-0.7			
	14-0.7			
	16-0.7			
	17-0.7			
	19-0.7			
Wire mesh	GJ-4 *			
	GJ-6			
	GJ-8			

* GJ-4 indicates a 4 mm diameter reinforcing mesh; 10-0.7 indicates a steel-plastic grid with 10-0.7 mm diameter wires in the tendon band.

**Table 4:** Summary of indoor test data.

Test Group	Test Number	Mesh Wire Tensile (kN)	Amount of Steel Used Per Unit Length of Mesh Wire (mm^3^)	Nodal Peel Strength (N)	Peak Mesh Load-Bearing Capacity (kN)	Contribution of Mesh Wire Carrying Capacity (%)
Steel-plastic geogrid	10-0.7	7.88	3.85	1224.38	53.00	48.04
	12-0.7	8.80	4.62	/	57.92	47.01
	13-0.7	10.10	5.00	/	/	/
	14-0.7	11.42	5.39	1154.56	64.92	40.61
	15-0.7	11.86	5.77	/	/	/
	16-0.7	12.12	6.16	1200.86	77.07	45.42
	17-0.7	12.35	6.54	/	82.38	47.65
	19-0.7	12.67	7.31	/	91.76	51.73
Wire mesh	GJ-4	8.53	12.57	988.35	34.40	28.81
	GJ-6	18.24	28.27	1728.46	39.98	15.66
	GJ-8	33.17	50.27		45.52	9.80

**Table 5:** Statistical table of profile spread data.

Mesh Type	Wire Mesh ^1^	Steel-Plastic Geogrid ^1^
Mesh size (m)	2 × 1	2 × 10.4
Number of meshes (pairs)	39.00	3.00
Mesh usage area (m^2^)	78.00	62.40
Overlap length (m)	0.20	0.20
Amount of anchors used	98.00	98.00
Support area (m^2^)	58.24	58.24
Average anchor use (/m^2^)	1.68 ^2^	1.68
Mesh utilisation (%)	74.67 ^2^	93.33
Overlap area (m^2^)	17.68 ^2^	4.16
Percentage of overlap area (%)	30.36 ^2^	7.14

^1^ This data is statistical for the selected mine area; different mine areas with different envelope conditions may differ from this table. ^2^ Average anchor use is the ratio of anchor use to support area; mesh utilisation is the ratio of support area to mesh use; overlap area is the area of the lap portion of the support plane; and Percentage of overlap area is the ratio of lap area to support area.

**Table 6:** Statistics on the time required for the construction of the site grid.

Type	Wire Mesh		Steel-Plastic Geogrid	
	Time (min)	Number	Time (min)	Number
Drilled holes	8	98	8	98
Installation of anchor rods	1.5	98	1.5	98
Mesh laying (m^2^)	1	78	0.5	62.40
Average lap time(m^2^)	6.5	17.68	5	4.16
Total time taken to hang the mesh in full section (min) ^1^	1123.92		983.00	
Average elapsed time (min/m^2^)	19.30		16.88	

^1^ This table of test statistics is tested for the mine site and varies from site to site and from environment to environment; the times quoted are for single-person work and are significantly reduced when more than one person works together.

**Table 7:** Test section cost statistics.

Type	Wire Mesh (Φ6)		Steel-Plastic Geogrid	
	Unit Price (CNY)	Number	Unit Price (CNY) ^1^	Number
Mesh (m^2^)	26.4	78	11.6	62.40
Number of anchor rods	34.2	98	34.2	98
Cost of lap material(m^2^)	46.2	17.68	25.4	4.16
Labour costs (min)	0.5 ^2^	1121.84	0.5	983.00
Total	6789.57		4672.60	
Average cost (/m^2^)	116.58		80.23	

^1^ CNY is the legal tender of the People’s Republic of China. ^2^ The price in this table is the statistical price of 2021, and different areas and different time will have some fluctuation; the labour cost is calculated at CNY 240 per day, working 8 h.

**Table 8:** Summary of key data from engineering trials.

Key Projects	Steel-Plastic Geogrid	Wire Mesh
Mesh utilisation (%)	93.33	74.67
Percentage of lap joints (%)	7.14	30.36
Average construction time (min)	16.88	19.30
Average construction cost (CNY)	80.23	116.58

## Data Availability

The data presented in this study are available on request from the corresponding author.
